# Nilotinib Versus Imatinib in Philadelphia Chromosome-Positive Chronic Myeloid Leukemia (Ph+ CML): A Systematic Review and Meta-Analysis of Randomized Controlled Trials (RCTs)

**DOI:** 10.7759/cureus.82631

**Published:** 2025-04-20

**Authors:** Fabeha Zafar, FNU Poombal, Laraib Ashraf, Divya Shivakumar, Diptish Wankhade, Rahul Winayak, Ghulam Mustafa Ali Malik, Shruti Sagar Mahapatra, Shasan G.C., Truc Huynh, Maneeth Mylavarapu

**Affiliations:** 1 Internal Medicine, Dow University of Health Sciences, Karachi, PAK; 2 Pathology, Nishtar Medical University, Multan, PAK; 3 Department of Medicine, CMH Lahore Medical College, Lahore, PAK; 4 Medicine, Kamineni Academy of Medical Sciences and Research Center, Hyderabad, IND; 5 Department of Medicine, Terna Medical College, Navi Mumbai, IND; 6 Bristol Medical School, University of Bristol, Bristol, GBR; 7 Department of Medicine, Dow International Medical College, Karacgi, PAK; 8 Internal Medicine, Srirama Chandra Bhanja Medical College and Hospital, Cuttack, IND; 9 Department of Medicine, Enam Medical College and Hospital, Dhaka, BGD; 10 Department of Medicine, Tan Tao University, Tan Duc E.City, VNM; 11 Department of Public Health, Adelphi University, Garden City, USA

**Keywords:** imatinib, nilotinib, philadelphia chromosome-positive chronic myeloid leukemia (ph+ cml), randomized controlled trials (rcts), systematic review and meta-analysis

## Abstract

Philadelphia-positive chronic myeloid leukemia (Ph+ CML) has witnessed significant advancements in treatment. Both nilotinib and imatinib have demonstrated potential in handling CML; however, a comprehensive evaluation of their comparative efficacy is crucial for optimizing treatment decisions. The primary objective of this study is to compare the rates of complete cytogenetic response (CCyR), major molecular response (MMR), and other clinical outcomes, including anemia, rash, and hyperbilirubinemia, in patients treated with either nilotinib or imatinib. An extensive literature search of reliable databases, including PubMed/MEDLINE, Scopus, clinicaltrials.gov, and Science Direct, was carried out to find the relevant literature. The subject headings and keywords searched were "imatinib", "nilotinib", “Ph+ chronic myeloid leukemia", and “Ph+ CML”. A statistical analysis was done using RevMan 5.4.1 (Cochrane 2020). Pooled effects were used to estimate the odds ratio (OR), hazard ratios (HR), and risk ratios (RR) with a 95% confidence interval (CI). I^2 ^statistics were used to assess for heterogeneity. A p-value ≤ 0.05 was considered statistically significant. A total of four studies with 1,045 patients were included in the study. Patients who received nilotinib had higher odds of achieving MMR at 12 months (OR 2.83; 2.12-3.79; p < 0.0001) and maintaining the MMR, i.e., durable MMR at 24 months (OR 2.72; 2.02-3.68; p < 0.0001) compared to patients on imatinib. Similar results were noted for CCyR at 12 months (OR 1.30; 0.60-2.81; p = 0.5). Overall, nilotinib was found to be superior due to better clinical outcomes, but adverse effects, like hyperbilirubinemia and rash, were significantly higher in nilotinib as compared with imatinib. With a paucity of quality studies conducted within this area, the clinical implications of our findings are limited. However, the use of nilotinib for Ph+ CML patients is an understudied area, with potential for clinical benefit over imatinib.

## Introduction and background

Philadelphia-positive chronic myeloid leukemia (Ph+ CML) has experienced remarkable advancements in treatment. The Philadelphia chromosome (Ph) results from a reciprocal translocation between the long arms of chromosomes 9 and 22, specifically at bands q34 and q11, represented as t(9;22)(q34;q11) [1-2]. Improvements in cytogenetic and molecular diagnostics have significantly impacted the management of CML, including diagnosis, prognosis, treatment, and monitoring. The discovery of the BCR-ABL1 fusion oncogene has facilitated the development of specific targeted therapies for CML patients, blocking tyrosine kinase activity. The therapeutic success of TKIs in CML is attributed to their specific inhibition of the BCR-ABL1 tyrosine kinase, the oncogenic driver of the disease [1]. Tyrosine kinase inhibitors (TKIs), which are the standard treatment for CML, improve survival rates despite potential side effects and risks of residual disease if treatment is discontinued, referring to the persistence of BCR-ABL1 transcripts, which can lead to a loss of molecular response and potential relapse, if treatment is discontinued [1]. Imatinib, a targeted therapy, represents a landmark in CML management because it addresses the abnormal protein structure produced by the Ph chromosome [3].

However, the issue of resistance and the need for improved outcomes have led to the development of second-generation tyrosine kinase inhibitors (TKIs), such as nilotinib [4]. Both nilotinib and imatinib are effective in managing CML. Nevertheless, we required a study to assess their comparative efficacies for optimized treatment decisions. Thus, we aimed to meet these criteria by conducting a meta-analysis of randomized controlled trials (RCTs) that compare the efficacy of nilotinib and imatinib in patients with Ph+ CML. This direct comparison allows for more precise estimates of relative effects, crucial for informing treatment decisions at the individual patient level. Furthermore, understanding the nuanced differences in efficacy and safety between nilotinib and imatinib is essential for optimizing treatment strategies, considering factors such as patient comorbidities, risk profiles, and quality of life. The primary goal of this study is to compare the rates of complete cytogenetic response (CCyR), major molecular response (MMR), and adverse effects in patients who received either nilotinib or imatinib.

## Review

Methods

**Figure 1:**
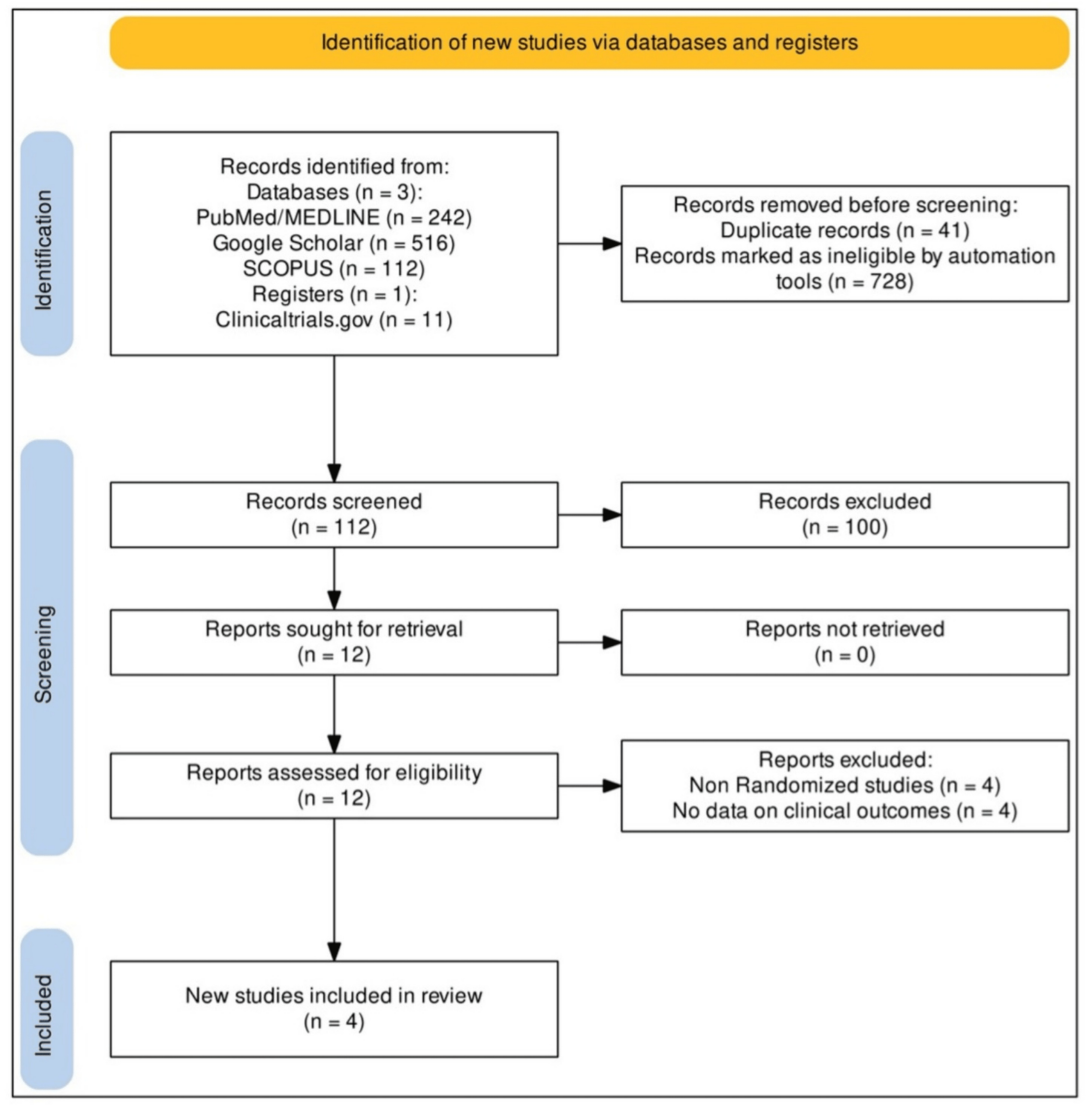
PRISMA (Preferred Reporting Items for Systematic Reviews and Meta-analyses) flow chart of the included studies

The PRISMA (Preferred Reporting Items for Systematic Reviews and Meta-analyses) and AMSTAR (Assessing the Methodological Quality of Systematic Reviews) guidelines were followed to conduct and report the study, respectively [5,6]. An extensive literature search of reliable databases, including PubMed/MEDLINE, Scopus, clinicaltrials.gov, and Science Direct, was conducted to find the relevant literature. The subject headings and keywords searched were "imatinib", "nilotinib", “Ph+ chronic myeloid leukemia", and “Ph+ CML”. The search terms were effectively combined using suitable Boolean operators. To ensure the extensiveness of the search, the references of the searched studies were also examined. The search strategy utilized for the study is outlined in Table 1. Studies containing data on the comparison between imatinib and nilotinib in Ph+ CML patients are included in the study. A detailed list of inclusion and exclusion criteria is outlined in Table 2. The study protocol was registered in the PROSPERO database (International Prospective Register of Systematic Reviews) (ID: CRD42024518896). Two reviewers, FZ and MM, independently screened the title, abstract, and full text. The conflicts concerning the screening were resolved by mutual consent. Figure 1 depicts the PRISMA flow chart outlining the study selection process [7].

**Table 1:** Search strategy

PubMed/MEDLINE
("Nilotinib") AND ("Imatinib") AND ("Philadelphia Chromosome-Positive Chronic Myeloid Leukemia" OR "Ph+ CML") AND ("Randomized Controlled Trial" OR “RCT”)
Google Scholar
Nilotinib OR Imatinib AND Philadelphia Chromosome-Positive Chronic Myeloid Leukemia OR Ph+ CML OR Randomized Controlled Trial
SCOPUS
- TITLE-ABS-KEY (Nilotinib OR Imatinib) AND TITLE-ABS-KEY (Philadelphia Chromosome-Positive Chronic Myeloid Leukemia OR Ph+ CML) AND TITLE-ABS-KEY (Randomized Controlled Trial)
ClinicalTrials.gov:
- Condition: "Philadelphia Chromosome-Positive Chronic Myeloid Leukemia" OR "Ph+ CML"
- Interventions: "Nilotinib" OR "Imatinib"
- Study type: interventional studies, observational studies
- Study results: randomized controlled trial

**Table 2:** Inclusion and exclusion criteria

Inclusion criteria	Exclusion criteria
Studies comparing nilotinib and imatinib in clinical outcomes in patients with Ph+ CML	No data on either nilotinib or imatinib or clinical outcomes in patients with Ph+ CML
Randomized controlled trials	Nonrandomized studies
Studies published in English	Studies published in languages other than English

Two reviewers, DS and P, independently evaluated the methodological quality of the included RCTs to ensure the appropriate quality assessment of the included studies. The risk of bias in the included studies was assessed using appropriate tools, including the Cochrane risk-of-bias 2 tool for RCTs. Statistical analysis was done using RevMan 5.4.1 (Cochrane 2020). A meta-analysis of pooled data was conducted using a random-effects model, due to the expected heterogeneity between studies, allowing for the incorporation of between-study variability into the analysis. Odds ratios (ORs) with relevant 95% CIs were reported. The choice of effect measure was dictated by the data available in the included studies. I2 statistics were used to assess heterogeneity, with thresholds of <25% indicating low heterogeneity, 25-50% indicating moderate heterogeneity, and >50% indicating high heterogeneity. A leave-one-out sensitivity analysis was performed to determine the studies that contributed to the heterogeneity. Publication bias was assessed using Egger's test and funnel plot analysis. The certainty of evidence for each outcome was assessed using the GRADE (Grading of Recommendations Assessment, Development and Evaluation) approach. We evaluated the risk of bias, inconsistency, indirectness, imprecision, and publication bias to determine the overall quality of evidence for each outcome. A p-value ≤ 0.05 was considered statistically significant.

Results

A total of four studies [8-11] with 1,045 patients were included in the study. A majority of the patients were males (59.9%). The patients who received imatinib (523) and nilotinib (522) were equally distributed. The baseline characteristics of the included studies are outlined in Table 3.

**Table 3:** Baseline characteristics of the included studies

Study IDs	Year	Total no. of patients	Mean age ± SD (in years)	Females (n%)	Nilotinib			Imatinib		
					Total no. of patients	Age (mean ± SD)	Females	Total no. of patients	Age (mean ± SD)	Females
NCT01275196 [8]	2015	267	40.6 ± 12.82	95 (35.6%)	134	41.5 ± 12.76	43	133	39.7 ± 12.26	52
NCT00519090 [9]	2011	6	54 ± 15.68	4 (66.7%)	2	69.5 ± 4.94	1	4	45.5 ± 12.06	3
NCT00471497 [10]	2019	565	-	249 (44%)	282	-	124	283	-	125
NCT00760877 [11]	2014	207	49.1 ± 13.16	71 (34.3%)	104	48.3 ± 13.26	33	103	49.9 ± 13.07	38

**Figure 2:**
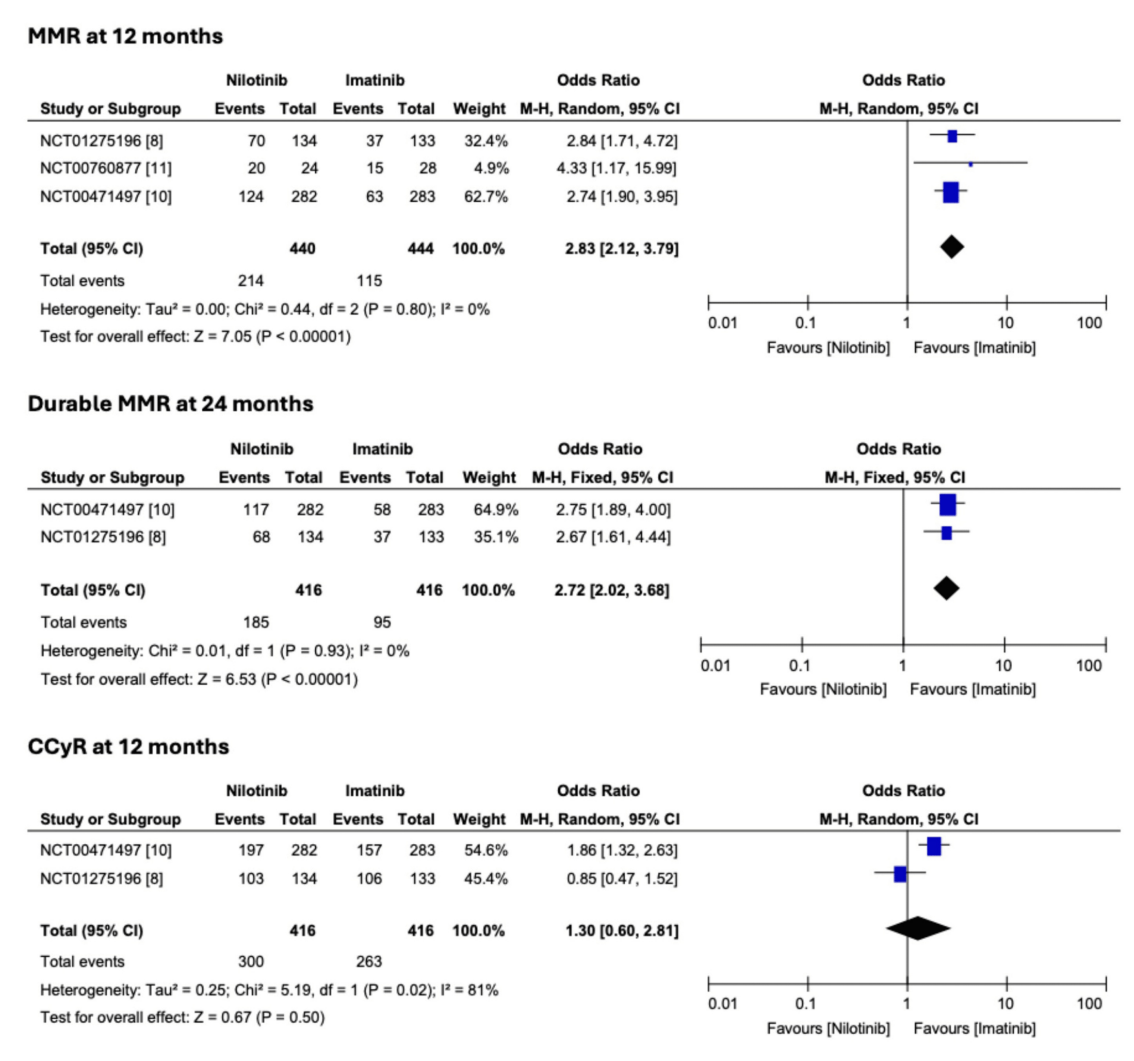
Comparison of nilotinib and imatinib on efficacy in Ph+ CML patients Ph+ CML, Philadelphia-positive chronic myeloid leukemia; MMR, major molecular response; CCyR, complete cytogenetic response

**Figure 3:**
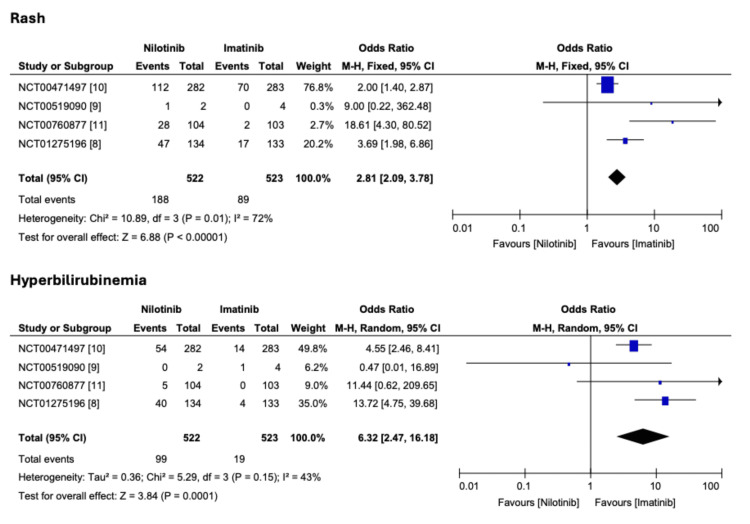
Comparison of nilotinib and imatinib on adverse effects in Ph+ CML patients Ph+ CML, Philadelphia-positive chronic myeloid leukemia

Patients who received nilotinib had higher odds of achieving MMR at 12 months (OR 2.83; 2.12-3.79; p < 0.0001) and maintaining the MMR, i.e., durable MMR at 24 months (OR 2.72; 2.02-3.68; p < 0.0001) compared to patients on imatinib. Similar results were noted for CCyR at 12 months (OR 1.30; 0.60-2.81; p = 0.5) (Figure 2). Regarding adverse effects, patients who received nilotinib had higher odds of rash (OR 2.81; 2.09-3.79; p < 0.0001) and hyperbilirubinemia (OR 6.32; 2.47-16.18; p < 0.0001) (Figure 3).

**Figure 4:**
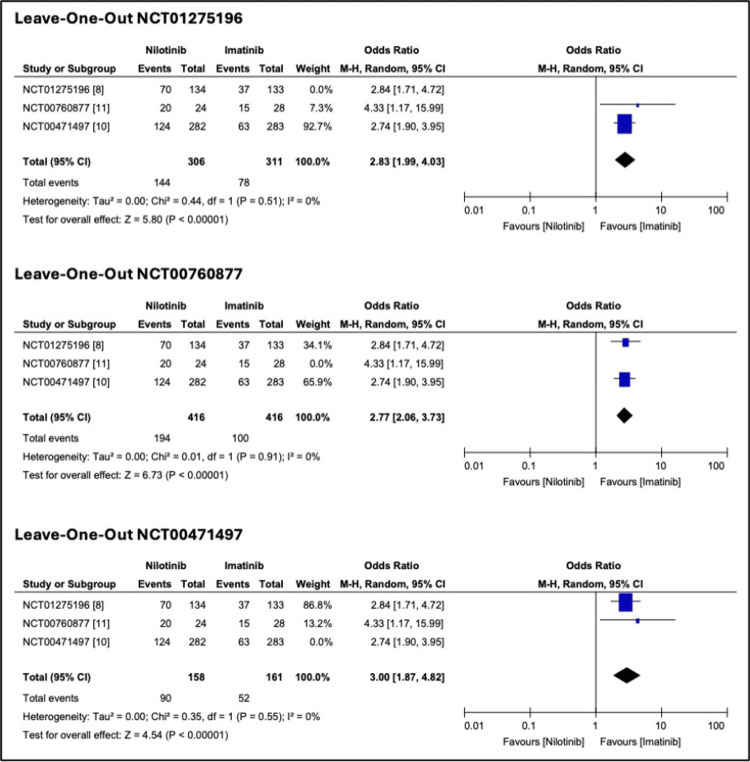
Leave-one-out sensitivity analysis for major molecular response (MMR) at 12 months

**Figure 5:**
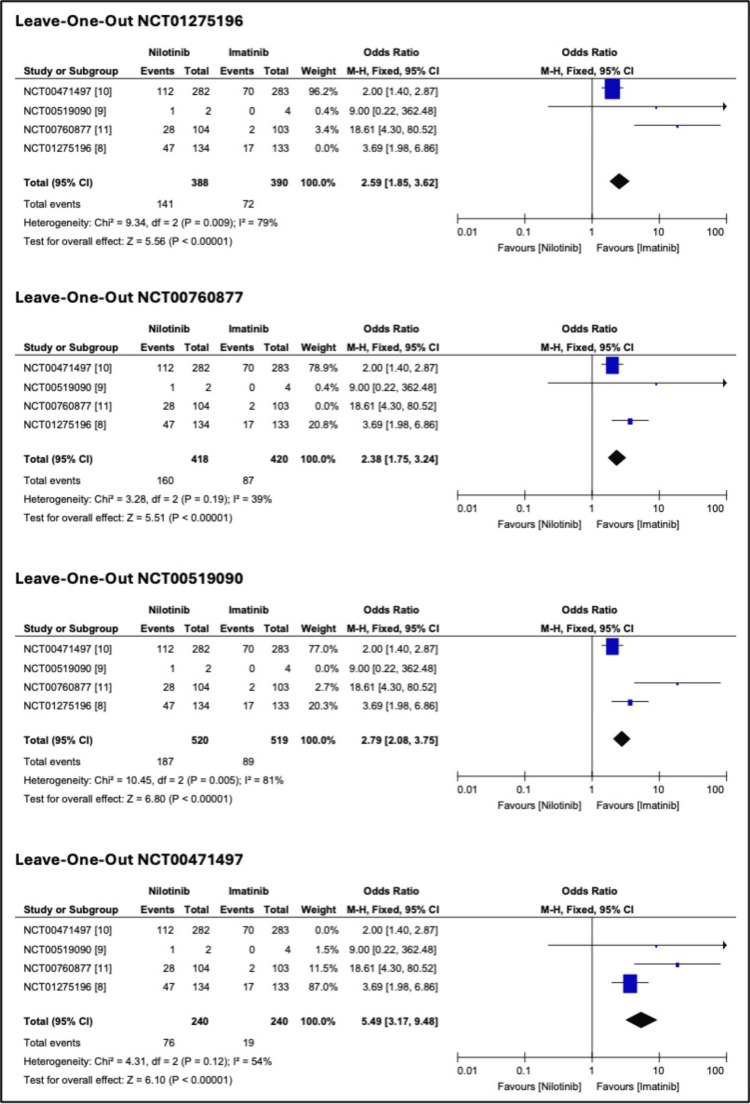
Leave-one-out sensitivity analysis for rash

**Figure 6:**
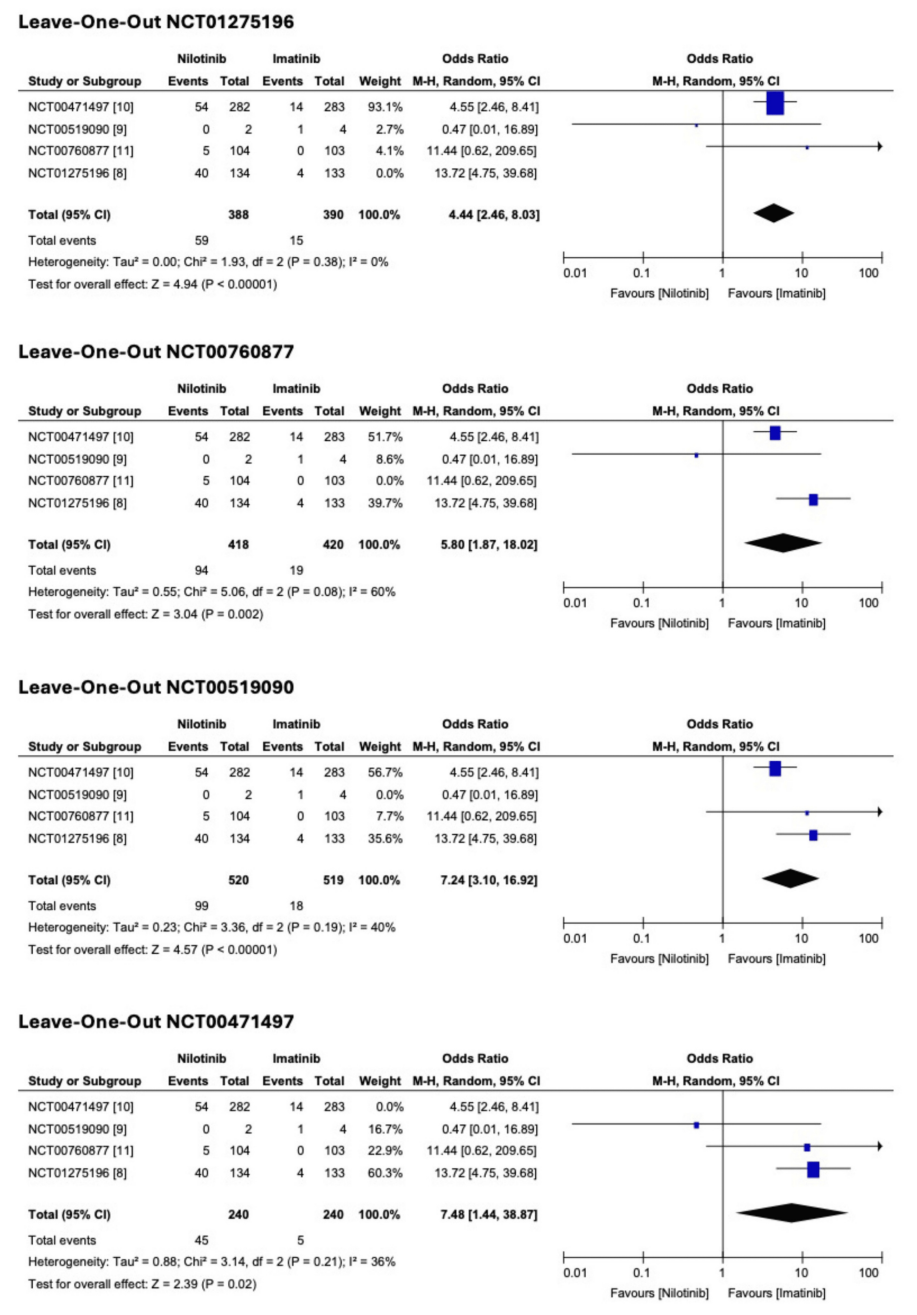
Leave-one-out sensitivity analysis for hyperbilirubinemia

A leave-one-out sensitivity analysis was performed to assess the robustness of the findings. The pooled ORs for MMR at 12 months (Figure 4) remained statistically significant across all iterations. For rash (Figure 5), the overall heterogeneity was 72%, with a notable decrease (39%) when Study 3 (NCT00760877) was omitted and a moderate reduction (54%) upon the removal of Study 1 (NCT00471497). For hyperbilirubinemia (Figure 6), the overall heterogeneity was 43%. The lowest heterogeneity (0%) was when Study 4 (NCT01275196) was removed. Publication bias was insignificant.

**Figure 7:**
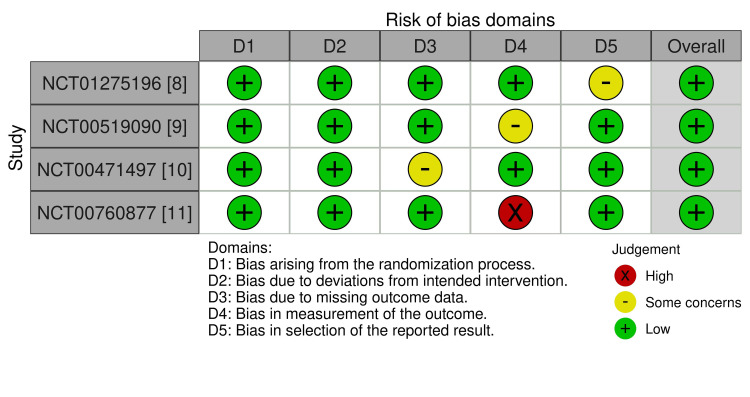
Risk-of-bias assessment of the included studies

**Figure 8:**
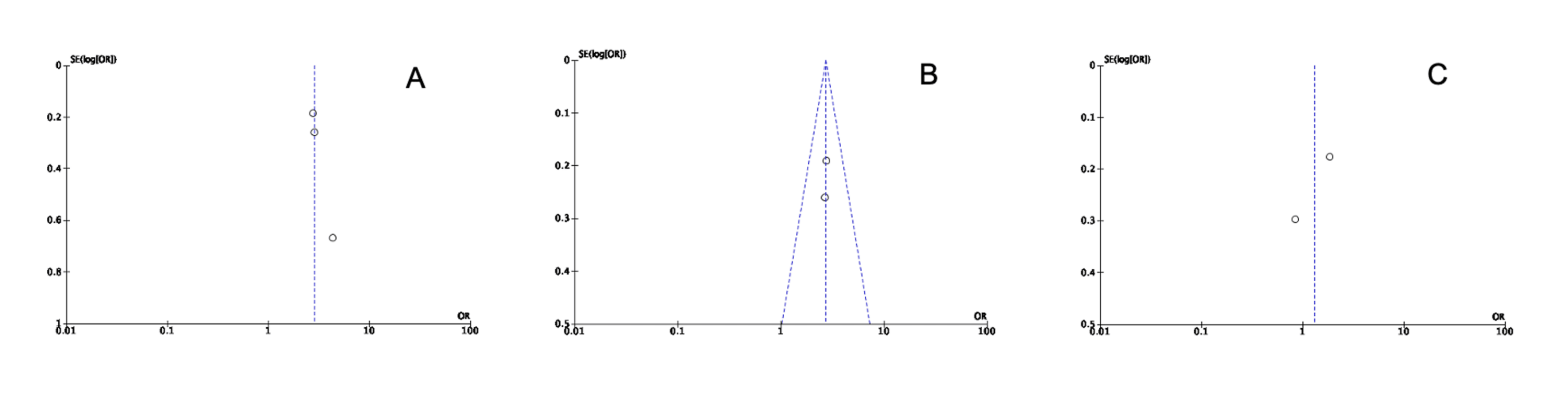
Publication bias assessment A. Major molecular response (MMR) at 12 months; B. durable MMR at 24 months; C. complete cytogenetic response (CCyR) at 12 months

Regarding GRADE assessment, the certainty of the evidence ranged from moderate (MMR at 12 months and durable MMR at 24 months) to low (CCyR at 12 months, rash, and hyperbilirubinemia) (Table 4). The risk of bias for the four included studies was evaluated, and all of the studies exhibited a low risk of bias regarding random sequence generation, allocation concealment, blinding of participants and personnel, and selective reporting. While three studies also displayed a low risk for blinding of outcome assessment, one study (NCT00760877) was unclear, which may introduce detection bias. Regarding incomplete outcome data, three studies were assessed with a low risk of attrition bias, while one (NCT00760877) presented a high risk, potentially affecting the validity of its findings. No additional sources of bias were identified across the included studies. Figure 7 summarizes the risk of bias assessment of the included studies [12]. Publication bias was insignificant, given the small number of included studies (Figure 8).

**Table 4:** GRADE (Grading of Recommendations Assessment, Development and Evaluation) assessment of the outcomes MMR, major molecular response; CCyR: complete cytogenetic response

Outcome: MMR at 12 months			
	Nilotinib	Imatinib	Effect estimate (95% CI)
Events	214	115	
Total	440	444	
Relative effect			OR 2.83 (2.12, 3.79)
GRADE assessment	Certainty of evidence		
Risk of bias	Some concerns due to risk of bias in some studies		
Inconsistency	Not downgraded: I² = 0%		
Indirectness	Not downgraded: direct comparison		
Imprecision	Not downgraded: precise estimate		
Publication bias	Not downgraded: no evidence of publication bias		
Overall certainty of evidence	Moderate		
Outcome: durable MMR at 24 months			
	Nilotinib	Imatinib	Effect estimate (95% CI)
Events	185	95	
Total	416	416	
Relative effect			OR 2.72 (2.02, 3.68)
GRADE assessment	Certainty of evidence		
Risk of bias	Some concerns due to risk of bias in some studies		
Inconsistency	Not downgraded: I² = 0%		
Indirectness	Not downgraded: direct comparison		
Imprecision	Not downgraded: precise estimate		
Publication bias	Not downgraded: no evidence of publication bias		
Overall certainty of evidence	Moderate		
Outcome: CCyR at 12 months			
	Nilotinib	Imatinib	Effect estimate (95% CI)
Events	300	263	
Total	416	416	
Relative effect			OR 1.30 (0.60, 2.81)
GRADE assessment	Certainty of evidence		
Risk of bias	Some concerns due to risk of bias in some studies		
Inconsistency	Downgraded one level: I² = 81%		
Indirectness	Not downgraded: direct comparison		
Imprecision	Downgraded one level: wide CI, non-significant result		
Publication bias	Not downgraded: no evidence of publication bias		
Overall certainty of evidence	Low		
Outcome: rash			
	Nilotinib	Imatinib	Effect estimate (95% CI)
Events	188	89	
Total	522	523	
Relative effect			OR 2.81 (2.09, 3.78)
GRADE assessment	Certainty of Evidence		
Risk of bias	Some concerns due to risk of bias in some studies		
Inconsistency	Downgraded two levels: I² = 72%, substantial heterogeneity		
Indirectness	Not downgraded: direct comparison		
Imprecision	Not downgraded: precise estimate		
Publication bias	Not downgraded: no evidence of publication bias		
Overall certainty of evidence	Low		
Outcome: hyperbilirubinemia			
	Nilotinib	Imatinib	Effect estimate (95% CI)
Events	99	19	
Total	522	523	
Relative effect			OR 6.32 (2.47, 16.18)
GRADE assessment	Certainty of evidence		
Risk of bias	Some concerns due to risk of bias in some studies		
Inconsistency	Downgraded one level: I² = 43%		
Indirectness	Not downgraded: direct comparison		
Imprecision	Not downgraded: precise estimate		
Publication bias	Not downgraded: no evidence of publication bias		
Overall certainty of evidence	Low		

Discussion

The findings of the studies suggest that nilotinib is superior to imatinib in terms of MMR for the treatment of CP-Ph+ newly diagnosed CML; however, the results of CCyR do not show statistical significance. The durable MMR is also 2.72 times higher in the case of nilotinib than imatinib. The analysis also demonstrated that adverse effects, such as rash and hyperbilirubinemia, were found to be significantly higher in the case of nilotinib than imatinib. The occurrence of anemia in the case of imatinib was slightly higher. Still, the significance could not be established due to the small sample size and only a few occurrences within the study subjects. At the time of its writing, our study represents the first meta-analysis investigating the efficacy and adverse effects of nilotinib versus imatinib for Ph+ CML patients. A number of meta-analyses were conducted that made a generalized comparison of second- and third-generation TKIs with imatinib, with a special reference to nilotinib for being the most effective TKI in terms of the outcomes.

The meta-analysis [13] by Vener et al., conducted in June 2020, included seven RCTs and compared imatinib with second- and third-generation TKIS for CP Ph+ CML patients. The study also included the overall survival, progression-free survival, and safety of all the TKIs. It demonstrated that the risk of cardiovascular events, fluid retention, pancreatic, hepatic, and thrombocytopenic adverse effects was significantly higher in nilotinib as compared to imatinib. Although the risk for cutaneous side effects of nilotinib, even in the dose of 400 mg BD, was not significant, as opposed to our study, wherein the development of rash was significantly adversely affected compared with imatinib. These observed differences could potentially be due to variations in patient populations and study design.

The meta-analysis [14] by Mealing et al., published in 2013, included eight RCTs. The study compared nilotinib and dasatinib against imatinib instead of individual comparisons between the drugs, and the description of the population included was not specific. The CCyR at six months and CCyR and MMR at 12 months were three and 2.4 times higher for nilotinib and dasatinib as compared with imatinib. Furthermore, there was no assessment of adverse effects. Overall, it could not help in suggesting the safety of the drugs. The meta-analysis [15] by Tang et al., published in 2019, included 34 RCTs and compared standard-dose imatinib with high-dose imatinib and newer TKIs, including nilotinib, dasatinib, and radotinib. Overall, the newer TKIs were superior in MMR at 12 months. Durable MMR was not analyzed, and no individual regard for any of the newer TKIs was made.

The network meta-analysis [16] by Signorovitch et al., published in 2013, compared the MMR of nilotinib and dasatinib versus imatinib for CP CML Ph + patients and included three ENESTnd trials of nilotinib versus imatinib. Nilotinib was associated with the highest MMR, with a 97% chance of being the most effective treatment based on MMR at 12 months. The adverse effects relating to any drugs were not included in any form. A few studies [17-19], including some case reports, have been conducted to estimate the occurrence of rash with nilotinib. These studies did not exclusively report rash in nilotinib but included all the TKIs or compared newer TKIs. Overall, the occurrence of rash in the nilotinib subgroup patients in both studies was significant. A clinical trial (NCT02108951) was conducted in Australia but could not continue because of slow recruitment.

Nilotinib is an inhibitor of the UGTA1A gene, which is involved in the metabolism of bilirubin, causing a buildup of bilirubin in the body by preventing its elimination [20]. Patients with UGTA1A polymorphism, when treated with nilotinib, can develop hyperbilirubinemia [21]. Our study also shows that hyperbilirubinemia was significantly higher in patients treated with nilotinib as compared with imatinib. However, the number of patients in our study who suffered from UGTA1A polymorphism was unknown. Therefore, testing patients for bilirubin levels or UGA1A polymorphism before commencing nilotinib could be a potential research area in the future. The average age of CML diagnosis is 64 years [22]. According to the ESMO guidelines, the major cause of mortality in CML patients is comorbidities. The cardiovascular adverse effects like coronary artery disease, acute coronary syndrome, cerebrovascular accidents, peripheral arterial disease, and arrhythmias with nilotinib are well known in Sicong li et al.'s meta-analysis, including 14 studies [23]. However, our analysis could not assess this adverse effect because the clinical trials had an insufficient sample size and data. 

Although it can be estimated that nilotinib's efficacy is superior, it is still unknown whether it is safe to be prescribed to large populations [24]. The ambiguity and difference in adverse effects in various meta-analyses could be a result of heterogeneity of populations and differences in the mean age groups included in the studies, prior treatments with other drugs, and the difference in the inclusion and exclusion criteria of the studies, for instance, the studies of this meta-analysis aimed to exclude patients having QT syndrome which may not have been excluded in other meta-analysis studies.

However, our study is not devoid of limitations. Firstly, the limited number of eligible studies restricted the comprehensiveness of our analysis. Secondly, our focus on specific adverse effects, namely rash and hyperbilirubinemia, may not fully capture the complete safety profile of nilotinib. Furthermore, a high risk of attrition bias in one of the included studies could potentially skew results if dropouts were differential between groups. In addition, unclear blinding of outcome assessment in the same study could have introduced detection bias, influencing outcome judgments. While other domains were generally at low risk, the possibility of undetected minor biases influencing overall evidence should be considered. These biases could have introduced some uncertainty in the results. Despite these potential biases, the overall quality of the included studies was good. Future research with a stronger focus on blinding and minimizing other potential biases is warranted to provide more robust evidence.

In addition, the heterogeneity of patient populations and study characteristics across the included trials could influence the generalizability of our findings. The limited number of studies could have also contributed to the observed heterogeneity. Finally, nilotinib's long-term efficacy and safety compared to imatinib require further investigation in larger and more diverse patient populations. These limitations underscore the need for a cautious interpretation of the results and emphasize the importance of future research to validate our findings and provide a more comprehensive understanding of the comparative benefits and risks of nilotinib and imatinib in treating CML. Furthermore, the impact of UGTA1A polymorphism on hyperbilirubinemia, at the current stage, is a hypothesis and needs to be further studied.

## Conclusions

While our meta-analysis suggests that nilotinib may offer superior clinical outcomes compared to imatinib for Ph+ CML patients, with moderate-certainty evidence for improved molecular response, this potential benefit is accompanied by an increased risk of hyperbilirubinemia and rash, for which the certainty of evidence is low. The limited number of eligible studies and the heterogeneity of patient populations warrant a cautious interpretation of these findings. Therefore, clinicians should carefully weigh the potential benefits of nilotinib against its risks, considering individual patient factors and monitoring for adverse events. Further large-scale, international randomized clinical trials are needed to confirm nilotinib's long-term efficacy and safety in diverse CML populations. These trials should comprehensively assess both efficacy and adverse effects to provide a more definitive evaluation of nilotinib's clinical utility and tolerability for Ph+ CML patients, enabling clinicians to make more informed treatment decisions based on a comprehensive understanding of the balance between potential benefits and risks.
